# Stochastic electromagnetic field propagation— measurement and modelling

**DOI:** 10.1098/rsta.2017.0455

**Published:** 2018-10-29

**Authors:** Gabriele Gradoni, Johannes Russer, Mohd Hafiz Baharuddin, Michael Haider, Peter Russer, Christopher Smartt, Stephen C. Creagh, Gregor Tanner, David W. P. Thomas

**Affiliations:** 1 School of Mathematical Sciences, University of Nottingham, University Park, Nottingham NG7 2RD, UK; 2 George Green Institute for Electromagnetics Research, University of Nottingham, University Park, Nottingham NG7 2RD, UK; 3 Institute of Nanoelectronics, Technical University Munich, Munich, Germany; 4 Centre of Advanced Electronic and Communication Engineering, Universiti Kebangsaan Malaysia, Bangi 43600, Malaysia

**Keywords:** electromagnetic fields, stochastic fields, propagation, Heaviside

## Abstract

This paper reviews recent progress in the measurement and modelling of stochastic electromagnetic fields, focusing on propagation approaches based on Wigner functions and the method of moments technique. The respective propagation methods are exemplified by application to measurements of electromagnetic emissions from a stirred, cavity-backed aperture. We discuss early elements of statistical electromagnetics in Heaviside's papers, driven mainly by an analogy of electromagnetic wave propagation with heat transfer. These ideas include concepts of momentum and directionality in the realm of propagation through confined media with irregular boundaries. We then review and extend concepts using Wigner functions to propagate the statistical properties of electromagnetic fields. We discuss in particular how to include polarization in this formalism leading to a Wigner tensor formulation and a relation to an averaged Poynting vector.

This article is part of the theme issue ‘Celebrating 125 years of Oliver Heaviside's ‘Electromagnetic Theory’’.

## 1. Introduction

Oliver Heaviside was a gifted engineer and a tenacious supporter of the *treatise* of J. C. Maxwell [1]. Along with George Francis FitzGerald and Oliver Lodge, he was part of a group of scientists later named ‘The Maxwellians’, who worked to develop an electromagnetic (EM) theory from Maxwell's explanation of Hertz’ experiments. Heaviside became known as the idiosyncratic genius [2] and maverick mind [3] of electricity for his practical intuition in understanding the wave guidance for wireless telegraphy, predicting a conducting layer in the atmosphere, later named after him and Arthur Edwin Kennelly, who formulated this idea independently at the same time [4]. The feasibility of long-distance wireless telegraphy had earlier been doubted by many scientists. Guglielmo Marconi pioneered wireless telegraphy and accomplished the first transatlantic signal transmission in 1902 using coupled resonant circuits as developed by Ferdinand Braun [5,6]. In 1909, Ferdinand Braun and Guglielmo Marconi jointly received the Nobel Prize for their groundbreaking contributions to wireless telegraphy. Besides his forward-looking activity in EM phenomenology, Heaviside gained a reputation as an important mathematician when he argued in favour of the use of vectors over quaternions, emerging in mathematical physics through the Cambridge fashion for Lagrangians [7]. Heaviside invented the vector notation to bring Maxwell's views closer to the more practical attitude of telegraphers as well as to relate the underlying symbols to intuitive geometrical operations [8, 5.5.3]. Heaviside's vector notation for Maxwell's equations has become standard, providing a balance in complexity and intuitiveness which makes it appealing for engineering. Further frameworks have been developed and applied to EM theory in the meantime which, unlike Maxwell's original formulation, provide a more intuitive insight such as the framework based on exterior differential forms [9,10]. Besides Heaviside's work on the generation and motion of wavefronts in open media and telegraphic lines, he soon focused on the definition of energy flux in electromagnetism. Thanks to his—geometry-based—vectorial notation, he derived an expression for **E** × **H** that made it clear that this quantity is related to the transport of energy. He achieved this result independently of Poynting, and even generalized it to include displacement currents [8, 5.5.3 and 5.5.4, pp. 196–199].

There is therefore a tradition in Maxwellian electromagnetics to reconcile theories with concepts involving the definition of EM energy and its transport vector. We shall take up this tradition, using the established Heaviside vector notation to extend the characterization of random scalar wave-fields to stochastic vector EM fields exploring the connection between correlation functions (CFs) and the Poynting vector. Furthermore, the use of Wigner functions is introduced to preserve the concept of directionality thus building on Heaviside's intuition finding regularities in the presence of complex field distributions. The Wigner function (WF) has originated in quantum mechanics [11,12] and has since found widespread applications in fields such as optics [13–15] and radio science [16].

Today, with the principles and models of electromagnetism well established, it is easy to forget the tribulations and controversy that the Maxwellians went through in the early days. One of the first episodes of this kind concerned MacCullagh's geometrical theory of optics and his formulation in terms of a quadratic Lagrangian describing the EM waves within the aether [17, p. 9], a theory dissmissed by Thomson and Stokes. Elastic theories of an aether based on Green's dynamical models were better regarded at that time in England. Stokes stated that MacCullagh's theory violated the conservation of angular momentum and so it was dynamically inadmissible [17, p. 10]. FitzGerald, who studied under MacCullagh at Trinity College Dublin, was reluctant to accept Stokes’ demolition of his alternative theory and put a serious effort into understanding Maxwell's *treatise*. Just when Stokes proved that Maxwell's theory was inadmissible as an elastic theory, FitzGerald concluded that Maxwell's theory was identical to MacCullagh's theory. But the acceptance of Maxwell's theory in the scientific community was still all but certain; this changed only when a young, self-taught, British telegrapher named Oliver Heaviside crossed FitzGerald's path to reshape the entire subject of electromagnetics. Heaviside, who never held an academic position and left school at 16 to work for a telegraphic company, dedicated his early efforts to the motion of EM waves in cables. Later, the generation and motion of EM waves in aether became a central theme in his studies. Inherently, Heaviside considered the concepts of electric and magnetic forces and fluxes central to the theory and preferred them over potentials. This was a key intuition in reducing the long list of Maxwell's original equations to a few equations constituting the *Maxwell's equations* of the EM theory as we know them today. Heaviside's interests were far-reaching to the point that he considered more complex motion scenarios besides guided and free waves, including the presence of nearby objects as well as wave confinement in enclosures. We will in the following analyse a small part of volume 1 of the *Electromagnetic Theory* series [7], where Heaviside mentions the *formation of a new regularity in the mean from complex fields in irregular geometries*. This is a clear precursor of the field of statistical electromagnetics, which is today pervasive in practical electromagnetic compatibility (EMC) and wireless communications scenarios. Importantly, Heaviside mentions the possibility of complex field distributions resulting from irregular sources, again, of paramount importance in modern EMC studies concerning the characterization of stochastic emissions from multifunctional digital electronics.

Modelling stochastic EM fields is necessary for improving the design of electronic devices, printed circuit boards (PCBs) and electronic systems taking into consideration their susceptibility to electromagnetic interference (EMI). Radiated EMI is caused by fast transients due to switching and information transfer processes within the electronic device. From an EMC perspective, these processes can be considered as being quasi-random, i.e. noisy. Power levels of the radiated field are in general low and emissions are spatially distributed over the PCB, while various hot spots may be identified. A widely used technique for characterizing emissions is near-field scanning (NFS) because of its high measurement accuracy and reliability [18–20]. This allows for using a canonical method in EM theory: by considering a source distribution enclosed within a virtual surface, one can compute the EM field in a source-free region outside this surface from amplitude and phase of the tangential field components on this surface by using Huygens' principle. For stochastic fields, however, numerical values of noise amplitudes cannot be specified by this method. On the circuit level, noisy signals can be described by energy and power spectra [21] and noisy linear circuits can be modelled numerically by correlation matrix-based methods [22–24]. In addition, network methods can be adapted for numerical modelling of EM fields [25] efficiently. Correlation matrix-based methods have been expanded in [26,27] to model noisy EM fields.

Another approach to modelling the propagation of field CFs is obtained by using a representation of the field in the combined space of position and wavevectors. The process is carried out through a representation known as the WF method [15], from which a ray-tracing-based approximation of the propagated CF can be derived [28,29]. Furthermore, in the propagation of CFs calculated from NFS of noisy fields, the connection between CFs and phase-space distributions to extract explicit directional information from the measurement process can be exploited. Interestingly, the method is able to include the transport of evanescent wave CFs as a leading order approximation of phase-space diffusion [30].

The concept of directionality was stressed by Heaviside when he was considering reflections from irregular geometries, envisaging new regularity for mean values for highly irregular fields. Representing EM fields in phase space goes beyond Heaviside's insight and leads to new insight in the form of finding the universal behaviour of completely randomized fields and including partial positional and directional information for geometries that are only partially irregular [31]. So far, we have treated the propagation of CFs of scalar field components in free space [29] and confined space [32]. In this paper, we discuss extensions of this formalism to include vector fields, which naturally leads to a Wigner tensor (WT). In previous work, predictions based on this WF approach have been verified in both the far field [29] and the near field [33]. NFS for field correlations in stochastic EM fields requires at least two field probes and has been addressed in [34–37]. The method has been validated experimentally in the context of cavity-backed apertures [29], Arduino printed circuit boards (PCBs) [38,39], and equipment-level enclosures [30].

## 2. From deterministic to statistical electromagnetic theory

Heaviside's masterpiece *Electromagnetic Theory* [7,40,41] contains a thorough analysis of two important properties of the electromagnetic wave (EMW):

— The electric and magnetic vector forces are subject to an effect of self-induction. Stress is made on this effect in pure (plane) EM waves, where *electric and magnetic forces have a constant ratio*, *electric and magnetic energies are equal*, *they have the property of being perpendicular to one another […] and their plane is in the wavefront, or the direction of motion of waves is perpendicular to **E** and to **H**. It is the direction of the flux of energy*.
— The EMW is constituted by force fields best described by *vectors*. Of particular importance is the introduction of the vector product as a tool for unveiling the principle of the *continuity of the energy flux*. It is remarked that *the principle of the continuity of energy is a special form of that of its conservation*, or Newton's *principle of the conservation or persistence of energy*. However, *in the ordinary understanding of the conservation principle, it is the integral amount of energy that is conserved, and nothing is said about its distribution or its motion. This involves continuity of existence in time, but not necessarily in space also. But if we can localise energy definitely in space, then we are bound to ask how energy gets from place to place.* As we will see later in the paper, the concept of lines guiding the energy flux was introduced by Heaviside independently from J. H. Poynting, and using the vector EMW notation.

Heaviside's perspective allowed for reducing the numerous equations introduced by Maxwell into four compact vector equations, as they are known today. An energy-based interpretation of EMW motion emerges by picturing its continuous flux that traverses a region of space from source to receiver. This interpretation is fully supported by mathematical tools from vector calculus, as opposed to quaternions put forward by Hamilton [7], and finds a direct connection to the directional energy flux analysis performed by Poynting. Inherently, it is the physics-based use of electric and magnetic field products connected to the energy flow that introduces the concept of directionality when waves transition through an unbounded medium. In this unique and imaginative reading of a new form of motion, inspired by cable studies of EMW, and consolidated by the tight interaction between the Maxwellians, modern EM theory finds its origins.

Heaviside was also keen to understand the diffusive nature of EMW energy across diverse media. Volume I of [7] contains a detailed discussion on the nature of EM wave transmission in analogy with diffusion processes in thermodynamics and mechanics. Special attention is devoted to the self-interaction mechanism between electric and magnetic fields, which makes up the propagation mechanism and determines its faster occurrence as compared to fluids.

It is probably the marriage between self-interaction and diffusion that lead Heaviside to think about the mechanism of EMW energy confinement within enclosed reflecting surfaces. The description of such a scenario is peculiar and deserves special attention as it is most likely related to a less known appetite of Heaviside for complex EMW distribution in irregular geometries underpinned by statistically isotropic waves giving rise to new forms of regularities, the *regularity of the mean*.

### (a) Characterization of stochastic sources

Electric current densities (or *Gaussage*) flowing through surfaces or volumes become a source of EMW that propagates in the surrounding space. Many of the ideas used below to predict such propagation in the context of complex or stochastic fields find precursors already in the work of Heaviside.

In Heaviside's idea of source operation, a central goal is *to find the form of the wavefront due to any collection of point-sources and trace the changes of its shape and position as it progresses* [7]. More specifically, he states that *when point sources are spread continuously over a surface to form a surface-source, we have a continuous wavefront from the first moment; or rather, two wavefronts, one on each side of the surface*. He continues by pointing out that *when we have once got a wavefront we may ignore the sources which produced it and make the wavefront itself tell us what its subsequent history will be*. Unilateral approximation and wavefront reconstruction is what can be achieved with the NFS method that will be explained later. The distinction between using the source currents explicitly, or ignoring them in favour of working directly with field measurements, is reflected in the two approaches to stochastic field propagation described in §2b.

Heaviside did not exclude the possibility of having, similarly to thermal diffusion, a diffusion of wavefronts originating from arbitrary sources in confined geometries and leading to complex EMW distributions. He realized that any localized perturbation will cause a distortion in both the space and time motion of waves. This perturbation can be described and predicted mathematically for elementary (point-)sources. When the source is extended and supports a complicated spatial current pattern, it can be described statistically, as depicted later in this section.

A natural step to take from the concept of energy defined by Heaviside is to consider a mean energy flux and Poynting vector through products of local, simultaneous fields. It can be shown that joint positional and directional information can be extracted from products of non-local and non-simultaneous fields. Discretely or continuously spread sources that are spatially extended, complex/irregular in their geometry, and stochastic in time, radiate wavefronts that have an irregular and stochastic structure. From any resultant (vector) field **F**(**x**, *z*;*t*), taking the product of fields and averaging in time to extract the typical field behaviour, leads to the *field correlation tensor (dyadic)*, that is,
2.1
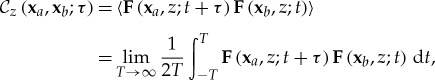
where the dyadic product is denoted **FF** = **FF**^*H*^ = **F**⊗**F**, with superscript *H* meaning conjugate transpose, while **x**_*a*_ and **x**_*b*_ are the (two-dimensional) transverse coordinates within planes parallel to the source and perpendicular to *z*, the outward normal to the source. Then using space–time stochastic magnetic fields yields **F**≡**H**, while starting from the electric field yields **F**≡**E**. In a Cartesian frame of reference **F** = [*F*_*x*_, *F*_*y*_, *F*_*z*_] the structure in (2.1) reads
2.2
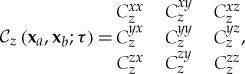
where the dependence on **x**_*a*_ and **x**_*b*_ and on *τ* in the entries has been omitted for compactness. Individual components of the correlation tensor (CT) (2.1) have been used in previous work on radiated emission in EMC, particularly using the experimental access to magnetic field components that is viable with commercial loop probes [29]. In the next section, we will describe how magnetic fields can be extracted accurately through scanning measurements in close proximity to the source. The experimental layout here gives direct access to the magnetic field components parallel to the source, *H*_*x*_ and *H*_*y*_, while the normal component *H*_*z*_ can be obtained both from the Gauss law for magnetism and from Faraday's law of induction [7]. An application of Love's equivalence principle has been proposed in [42], where the concept arises of a Huygens box embracing a radiative emission source for EMC tests. Only tangential magnetic fields on the box surface are needed to find the radiated emissions from the source by defining the equivalent surface current density **J**_*s*_ = **n** × **H**, with **n** the outward normal to the surface of the Huygens box. Then curl equations with the forcing term **J**_*s*_ are used to calculate the tangential components of the electric field. Alternatively to measuring magnetic fields, experimental set-ups may also measure electric fields. Notable examples are the sleeve dipole used at UMIST [42] and the atomic field probe recently conceived at NIST [43]. Therefore, defining the coherence tensor in terms of either electric or magnetic fields allows one to create a model of the source based on laboratory measurements. Combining electric and magnetic fields, it is also possible, in principle, to form hybrid *magneto-electric* CFs. In fact, any combination for which there is an explicit relation between the resulting coherence tensor and the Poynting vector, which describes the local direction of the energy flow [44], allows propagation of emissions from the source. We illustrate this explicitly later in this section, by showing how the coherence tensor can be used to devise a wave-dynamical phase-space representation from which both the energy flow vector and the local energy density can be extracted self-consistently.

In the frequency domain, this field–field CT is represented by the Fourier transform
2.3
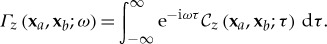
The averaging in (2.1) produces a function that is independent of the time reference in the case of stationary stochastic fields. Hidden in the CF is significant information on the physical structure of the random emissions. However, the spatial representation (2.3) does not give simple access to this in terms of a clean physical interpretation. Following arguments similar to those employed in semiclassical quantum mechanics for the single-particle density function [13], it can be argued that both positional and directional properties can be extracted from the CT through the WT [28].

The WT has a direct connection with the CT and transforms its entries to WFs that represent vector wave components' functions on phase space, combining both position and direction of propagation. More precisely, the WT is defined as
2.4
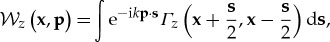
where *k* is the constant wavenumber coordinates (**x**, **s**) related to (**x**_*a*_, **x**_*b*_) by the transformation
2.5
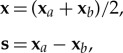
so that **x** is the average position and **s** is the difference in positions of a pair of measured fields. More explicitly, **s** = (*s*_*x*_, *s*_*y*_) represents, in the NFS of planar sources, the in-plane displacement (for fixed *z*) between measurement positions. The conjugate momentum vector **p** = (*p*_*x*_, *p*_*y*_) in (2.4) takes the geometrical meaning of the components of the wavevector parallel to the source plane, normalized so that
2.6
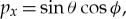

2.7
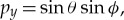
and |**p**| = sin*θ*, where *θ* is the angle of the ray with respect to the outward normal.

A useful property of the WF is that it allows for treating position and momentum variables symmetrically, so that (2.4) can also be obtained from
2.8
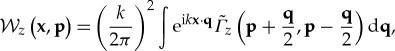
where coordinates (**p**, **q**) are similarly obtained from (**p**_*a*_, **p**_*b*_) through the rotation
2.9
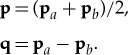
The momentum representation of the CF (2.3) is given by the double partial Fourier transform, defined in the frequency domain by
2.10
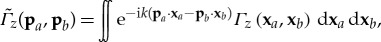
with **p**_*a*_ and **p**_*b*_, respectively, denoting the variables conjugate to **x**_*a*_ and **x**_*b*_: one similarly finds that (**p**, **q**) are respectively conjugate to (**s**, **x**). It is evident from (2.10) that phase-space methods are analogous to plane wave-based near-to-far-field transformations.

From the WT, an inverse Fourier transform retrieves the CT, in either a position or in a direction basis through
2.11
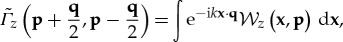

2.12
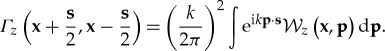
We now show that a direct connection between the Poynting vector, expressing the directional flow of energy across a predefined surface, and phase-space tensors, can be found through the CT of solely electric (magnetic) fields; see the Appendix for details. The starting point is the partial inverse Fourier transform introduced in (2.10) of both the electric and magnetic fields
2.13
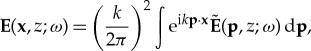

2.14
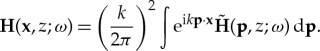
We now write the Poynting vector for a partially coherent field as [14]
2.15
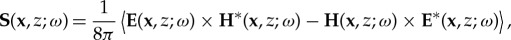
where 〈 · 〉 denotes an ensemble average over field realizations. Next, using Maxwell's equations in momentum space, along with the definitions of the CT in (2.3) and the WT in (2.4), we get (see Appendix)
2.16
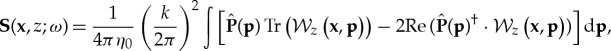
where 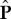 is a vector operator defined in (A 9) and 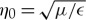 is the free-space impedance. This gives a direct relation between the Poynting vector and the WT and a similar expression holds, with the substitution 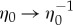, for the corresponding magnetic CT. Note that this relation holds in the non-paraxial regime for fully vectorial EM fields and can therefore be used to characterize stochastic near fields. Inherently, the first term in (2.16) has been found by Keller *et al.* in the context of waves propagating in weakly random media [45], while the additional terms have been found in [14] using a different formalism. The second term (see Appendix) is believed to be important in the near field of stochastic sources as it is non-zero only for non-plane wavefronts, i.e. it vanishes for plane wave-like EM fields, whence we expect it to be less important in the far-field region beyond the stochastic source. We remark also that the Poynting theorem, which establishes a relation between the rate of change of the energy density and the Poynting vector, can be used to derive a continuity equation for the Wigner representation of EM waves [45].

To conclude this section, we point out that a NFS of two rectangular magnetic field components tangential to the source is enough to have a full reconstruction of the average Poynting vector from the WT. Other forms of the Poynting vector can be used in connection with the WT of partially coherent fields, which offers an alternative representation to directional fields used to find a paraxial equation for the field intensity [46].

### (b) Transport of stochastic fields

We now review two approaches to propagate the source CT, a first one constructed through the intermediate representation based on the WT from NFS measurements [29], and a second one based on the method of moments (MoM) [27]. Once partially coherent stochastic fields radiated by a complex spatio-temporal source are characterized in terms of the WT, a phase-space transport equation is used to advance wave densities along their positional and directional characteristics. According to Liouville's theorem, the flow of scalar wave densities such as radiance is conserved along phase-space paths. It has been shown in a different context that this translates into a particularly simple law to advance WFs across finite regions in homogeneous media. More precisely, the WF of in-plane fields near a source can be translated to WFs beyond the source by a simple linear mapping that represents a sheared motion in phase space [13,15]. This has been obtained as a leading order approximation from the solution of both Helmholtz [28] and vector wave equations [29]. Briefly, the exact solution is obtained by representing the second Green identity in **p** space and applying the measured in-plane field from NFS as boundary data. In the context of vector EM fields, the Stratton–Chu boundary integral equation is solved in the *z*-plane [47] in the momentum **p** basis, and the free-space propagator for individual field components can be derived exactly. The propagated CF at *z* > 0 is thus obtained by using the fields measured at *z* = 0 as boundary conditions. An exact propagator follows for the field–field CF, from which the corresponding WF can be derived. Recasting the source CF obtained from boundary data into a source WF, an exact transport operator is found in integral form, which can be approximated at leading order with a Frobenius-Perron transport equation. This procedure has been applied to scalar fields, i.e. to a single component of the CT (2.3) and for the WT (2.4) [29, eq. (26)]. More specifically, individual tangent vector components of the in-plane magnetic field have been measured and used to guide and verify the approximate transport equations [29, fig. 9]. Starting with the source correlation in momentum space, calculated from boundary data, propagation along the normal direction to the source is described by the propagator
2.17
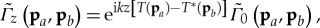
using the notation of (2.9) and where
2.18
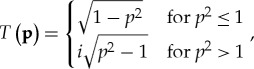
and *p* = |**p**|. The transport equation for the WT can then found by inserting (2.17) into (2.8)
2.19
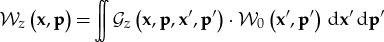
with a kernel given by the dyadic operator
2.20
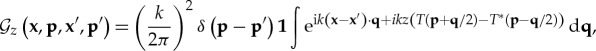
where **1** is the unit dyad, from which (2.21) can be written as [33]
2.21
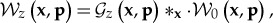
where *_x_ denotes the convolution operation acting only on the spatial variable **x**. It has been shown in [28,29] that 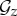 can be simplified through a ray-based approximation for spatial variations in the source correlation that are on a scale that is larger than the wavelength. The exponent in (2.20) can be approximated by expanding in **q**, to yield the representation of a Dirac delta function for propagating waves,
2.22
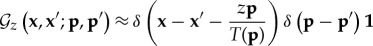
and an exponential damping for evanescent waves
2.23
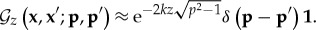
Finally, using (2.22) and (2.23) in (2.21) yields
2.24
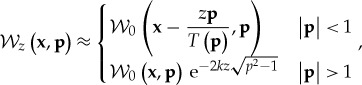
thus leading to a ray-tracing approach for propagating waves and to a **p**-dependent damping rate for evanescent waves. In the cases of proximity to the source or of low-frequency emissions, the evanescent component may be significant, if not dominant. Then the significance of large momenta going well beyond the leading order in (2.24) motivates an asymptotic calculation for high **p** leading to
2.25
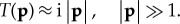
It has been found that the effect of the convolution on evanescent waves is twofold: there is a **p**-dependent decay combined with a diffusion in **x**. It is therefore found that the propagator can be derived conveniently by using the WT as an intermediate representation [29, Eq. (22)]. This approximation of the transport rule for WTs is obtained explicitly by retaining the leading order of the series expansion of the exponent of 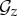 in (2.21) [28,48]. The propagated CT can be retrieved in a configuration space by an inverse Fourier transform as depicted in [29]. Interestingly, the average scalar EM field intensity can be obtained from the transported 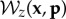 in the far field, which has the physical meaning of a (local) average radiation pattern from the statistical source. Used in the Poynting vector, the energy flow takes a particularly simple form in this free-space approximation, propagating field intensities along straight lines, with a tangent vector given by **p** from the source plane to the observation plane at *z*. The propagation rule for an in-plane CF offers an analogue to NF transformation of fields radiated by deterministic sources such as antennas [49]. We now describe an alternative propagation method using the MoM.

Heaviside pioneered the use of vector electric and magnetic potentials. Their use has become standard in EM theory and offers a convenient mathematical framework on which to calculate the radiated fields from a source electric **J**_*e*_ and/or magnetic **J**_*m*_ surface current density. The Poynting vector allows for creating equivalent electric currents from in-plane tangential magnetic field components, 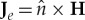. In the presence of deterministic sources, an equivalent distribution of electric dipoles can be reconstructed from an equivalent surface current density by back-propagating near fields. Having access only to field–field CFs, the same philosophy can be used with stochastic sources to derive propagated CF by forming a stochastic current–current CF [50].

It is shown in [26,27] that the source-field dyadic Green functions can be introduced through the vector potentials to obtain the field dyadic
2.26
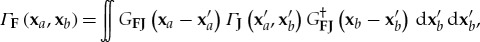


describing the correlation of the field **F** at points **x**_*a*_ and **x**_*b*_. The field dyadic *Γ*_**F**_(**x**_*a*_, **x**_*b*_) is defined in (2.3) and the current dyadic, related to the correlation of the the surface current density, is defined as
2.27
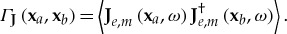
The twofold integral (2.26) can be treated analytically or the MoM [51] can be used to convert the field problem into a network problem and can be solved by subsequently applying network correlation matrix methods [22–24] as shown in [26,27]. By expanding the field and current densities in a vectorial basis
2.28
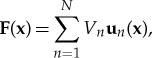

2.29
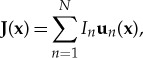
and substituting (2.28) and (2.29) in (2.26) and (2.27), we obtain a set of algebraic equations
2.30
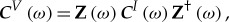
where we define *impedance* matrix elements through
2.31
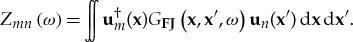
Having introduced the generalized voltage and current vectors, in (2.30)
2.32
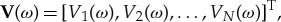

2.33
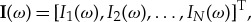
we have defined voltage–voltage
2.34
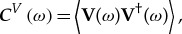
and current–current
2.35
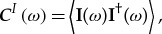
correlation matrices. Finally, the propagated field–field correlation dyadic (2.12) is retrieved in the position basis function as
2.36
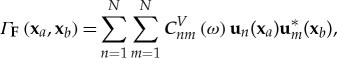
which can be compared with the propagated correlation obtained with the WT intermediate representation.

When detected by probes, EM fields are perturbed by the probe structure itself. As explained in [50], a more subtle effect in NFS concerns the coupling of probes with the surface current density distribution flowing at the source surface. A correction factor can be evaluated by either dedicated experiments or simulations, which can be used to predict the transfer impedance between the field incident on the probe and the voltage read from its port [52]. For statistical sources, it is therefore natural to consider correcting entries of the CT involving the correction factor. Nevertheless, it is shown through plane-wave expansion [53] and operator theory [50] that the propagation of probe voltage–voltage CFs is equivalent to the propagation of field–field CFs. A proposal for standardization of near-field measurement of stochastic EM fields led by the European COST Action 1407 has been initiated in [54]. This initiative has resulted in an IEEE standard proposal being specified for single-probe, dual-probe and multi-probe scanning systems. The measurement methods are compared in terms of their RF, accessible resolution, reliability (including mechanical stress) performances and test time for industrial deployment. The amount of data recorded in two-point measurements required for the characterization of stochastic EM near fields can be reduced considerably by principal component analysis [55–57].

The Green function and MoM methods also have been extended for application to cyclostationary stochastic fields [58–60]. Areas of application are the modelling of the EM interference radiated by digital circuitry inside the system and also into the environment, where the period of the cyclostationary EMI is given by the clock frequency of the digital circuits.

### (c) Statistical description of confined fields

The characterization of stochastic fields operating *in vacuo* is important in having an energy-based model of complex sources. It is then natural to include environmental effects in the propagation of CTs. Proximity of objects and/or source confinement, such as for a PCB inside a shielding enclosure, generates reflected waves, which interfere with free-space waves to create complicated distributions of the EM field. This phenomenon is enhanced inside resonant cavities. In irregular cavities, energy is diffused and localization is reduced at high frequencies. Heaviside's analysis of equilibrium radiation has strong precursory elements at the basis of modern statistical electromagnetics. The discussion in [7, sec. 186] starts from the realization that *… a perfectly conducting screen enclosing a dielectric region supporting electromagnetic disturbances, keeps in their energy, which remains in the electric and magnetic forms, and if there be no source of energy present, the total energy remains constant*. Then it continues towards the definition of the concept of resonance, which develops *the very rudimentary case of a plane wave running to and fro between parallel plane reflecting boundaries, without the slightest tendency to change the type of the vibrations*, for which *there is no necessary tendency for the initial state […] to break up and fritter down into irregular vibrations*. The subsequent sentence intentionally spoils this intuitive picture through acutely observing that *there does appear to be a general tendency to this [irregular] effect, when the initial states are not so artfully selected as to prevent it happening. Even when we start with some quite simple type of electromagnetic disturbance, the general effect of the repeated reflections from the boundary (especially when of irregular form) and the crossing of waves is to convert the initial simplicity into a highly complex and irregular state of vibration throughout the whole region*. More importantly for the case of statistical sources, Heaviside continues by saying that this *irregularity* occurs *if the initial state be itself of an irregular type, when it is tolerably clear that the irregularity will persist, and become more complete*. Heaviside's reasoning does not stop here and furthers the investigation by assuming a fully developed, *extreme*, irregularity and arguing that *the very irregularity gives rise to a regularity of a new kind, the regularity of averages. The total [average] energy, which is a constant quantity, will be half electric and half magnetic and will be uniformly spread throughout the enclosure, so that the energy density (or energy per unit volume) is constant. As regards the [electrical] displacement and the [magnetic] induction, they take all directions in turn at any one spot, quite irregularly, but so that their time-averages show no directional preference*. Invoking the variability in amount and direction of the flux of energy, expressed by the Poynting vector, Heaviside anticipates an interesting calculation to perform *[…] in virtue of the constancy of the mean density and the preservation of the normal state by constant exchanges of energy, there is a definite mean energy flux to be obtained by averaging results. This mean flux expresses the flux of energy per second across a unit area anywhere situated within the enclosure*. Letting *[…] the mean density of the energy be *U* […]*, and fixing *[…] attention upon a unit area, *A*, […]*, the flux of energy through *A* is considered *under different circumstances*. The first one regards the overall energy moving at the same speed *v*, *[…] as in simple plane progressive waves, and the direction of its motion were perpendicular to the fixed unit area *A*, then the energy passing through it would belong to a ray (or bundle of rays) of unit section, and the energy flux would be *Uv* […]*. However, Heaviside points out that *[…] this is impossible, because energy would accumulate on one side of *A* at the expense of the other. The next approximation, to prevent the accumulation, is to let half the energy go one way and half the other; still, however, in the same line. This brings us down to 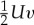. To go further, we must take all possible directions of motion into account.* In order to introduce an additional approximation based on multiple directions of wave motion, we need to imagine the ray *[ … ] to make an angle θ with the normal to A [ … ]. Considering this reduction of the ray energy, the true flux through the area A is therefore the mean value of Uv* cos *θ* *for all directions in space assumed by the ray. Now the mean value of* cos *θ* *for a complete sphere is zero, and therefore the mean flux through A is zero. This is right, as it asserts that as much goes through one way as the other. To obtain the amount going either way we must average over the hemisphere only. The mean value of* cos *θ* *is then* 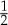. *But we are only concerned with half the total energy, or* 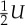, *when we are confined to one hemisphere. Consequently, we have*
2.37
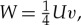

*to express the flux of energy W per second each way through any unit area in the enclosure.* This result is extremely important as it anticipates early studies on random EM fields in mode-stirred enclosures and reverberation chambers, which are at the basis of modern statistical electromagnetics [61]. Interestingly, Heaviside remarks that the result in (2.37) can be obtained following an alternative procedure, which is very similar to the earlier argument, but whose starting point is *to divide the original ray of unit section along which the flux is Uv into a very great number n of equal rays of unit section, each conveying* 1/*n* *part of the same, and placed at such inclinations to the normal to A that no direction in space is favoured. This amounts to dividing the surface of a sphere whose centre is that of the area A into n equal parts, the centre of every one of which defines the position of one of the n rays. Any ray now sends* (*Uv*/*n*)cos *θ through A per second. Now sum this up over the whole hemisphere and the result is W* [ … ] in (2.37). More explicitly, *in the limit, when n is infinitely great, we have*
2.38
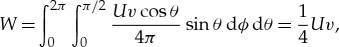

*as before.* The division of energy into partial rays has a remarkable analogy with the random plane wave hypothesis used to explain field fluctuations in reverberation chambers [62]. Having obtained this fundamental result, Heaviside goes on to point out analogies with previous investigators in thermodynamics, where it would also appear *[…] that the result is general, and is independent of sources of heat, and of the emissivity and temperatures*. Nevertheless, it is implicit that the fraction of radiation absorbed inside the cavity would be compensated by the same amount of emission, thus implying the presence of a source maintaining the extreme EM field state. Further considerations are put forward by Heaviside in [7, Sec. 187 and Sec. 188] concerning the mean pressure of radiation and the analogy between emissivity and temperature. Once again, results offer a precursor to developments in statistical electromagnetics, where a strong analogy with thermodynamics concepts is used to obtain average quantities, as summarized in [63]. Although Heaviside's intuition suggested that the conversion of simple sources into a *highly complex and irregular state […] cannot happen universally*, advancement of statistics as well as modern wave chaos—the study of wave systems whose classical, high-frequency asymptotics, analogue supports a chaotic dynamics – achieved a deeper understanding of the distribution of spectral eigenvalues of irregular systems, thus defining universal laws through random matrix theory (RMT) [64,65]. The random plane wave hypothesis is also supported by phase-space studies of chaotic systems, from which we now understand that the *extreme state* defined by Heaviside can be achieved by considering the field amplitude statistics governed by a universal Gaussian probability distribution: this offers one further example of *a regularity of a new kind*. The WF of a maximally entropic EM field state is uniform in phase space [31]. More modern statistical theories have been formulated to characterize average energy and probability density functions of irregular cavity fields, some inspired by the reverberation chamber [62,66,67], some inspired by wave chaos in confined microwave billiards [64,65]. However, the transition between regularity and irregularity is not sharp and in practical propagation scenarios, especially in wireless communication studies, confining geometries are not completely irregular as they can present flat walls facing each other, thus leading to mixed regular-irregular phase-space structures. The WT offers a valuable way to characterize the development of such a structure from partially coherent and partially polarized sources radiating inside partially irregular environments. A recent approach called the dynamical energy analysis (DEA) has been proposed to transport phase-space densities on triangular meshes [68,69]—similar to those employed in the finite-element method (FEM)—through the Frobenius-Perron operator. DEA is obtained as an asymptotic limit of a wave transfer operator for field–field CFs [70,71]. Taking the WT entries of (2.4) in the short-wavelength limit upon asymptotic expansion, we obtain at leading order that the transported average WT takes the form
2.39
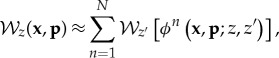
whose elements are the solution of the stationary Liouville equation used in the DEA method [69]. In (2.39), the linear phase-space flow *ϕ* is dependent on the chord connecting the point *z* to the point *z*′ at the boundary. This is the underlying ray-tracing scheme on which the WF develops wave effects. Specific scenarios have been studied to test the propagators that we have derived, including a fully chaotic quantum map [32], which serves as a prototype of the propagation in a diffuse environment such as reverberation chambers.

## 3. Experiments on stochastic electromagnetic fields

We validate the propagators of §3b through laboratory experiments conducted at the George Green Institute of Electromagnetics Research (GGIEMR), University of Nottingham. A source CF is obtained from measured magnetic fields of stochastic EM fields, see [29].

### (a) Experimental set-up

**Figure 1.**
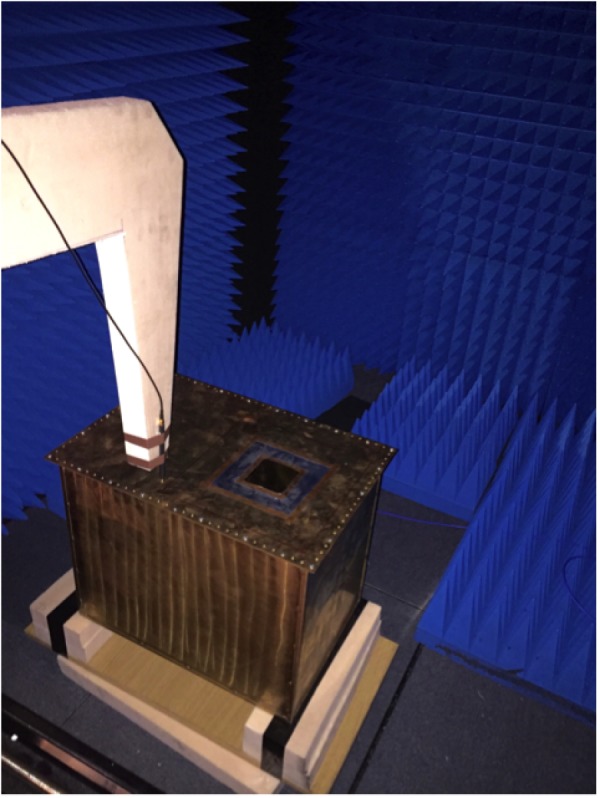
Loop probe moved above the cavity-backed aperture by the scanner system.

The WF-based approximate propagator in (2.24) will be compared to the exact MoM-based propagator (2.36) and experiments. A one-probe 3-D scanning system is used to perform measurements of a single magnetic field component radiated from the DUT in figure 1. The DUT consists of a metallic brass cavity with a 0.8 m × 0.8 m aperture on the lids shown in figure 1. A metallic rotating stirrer which is driven by a stepper motor is placed inside the cavity to mix and randomize the EM field radiated from the aperture. The source of the radiation is a monopole inside the cavity, a metallic rectangular enclosure of dimensions 1 m × 1 m × 0.5 m. The monopole is a loop antenna, Langer EMV-Technik RF R50-1 magnetic field probe, connected to an Agilent E5062A vector network analyser (VNA). Both magnitude and phase of the coupling between the monopole and the probe, *S*_21_ measurement, at a frequency of 3 GHz, are captured at each scanning position. The phase reference provided by the VNA makes it possible to calculate a CT by performing a single-probe scan over the source plane.

The scanner system is then placed inside an anechoic chamber to minimize external interference while being controlled using a LabVIEW software on a PC from outside. We perform a near-field scan close to the aperture: the magnetic field is recorded across a dense grid of spatial points by moving a loop probe over the scanning plane. The experiment is done at a fixed frequency *f* = 3 GHz (*λ* = 0.1 m).

The scanning plane size is 0.3 m × 0.3 m with 0.005 m scanning steps yielding 60 × 60 scan points per plane. The measurements were carried out for two scanning planes at heights of *z* = 0.01 m and *z* = 0.10 m above the source plane. The measurements were repeated for 36 different paddle positions with a 10-degree rotation step to create an ensemble of fields. Measurement for one paddle position on both scanning planes will capture the VNA transmission parameter *S*_21_ for a total of 2 heights × 60 points × 60 points =7200 measurement points. For 36 paddle positions, 259 200 data points are captured and this will provide some difficulty in processing of the data.

### (b) Results

**Figure 2.**
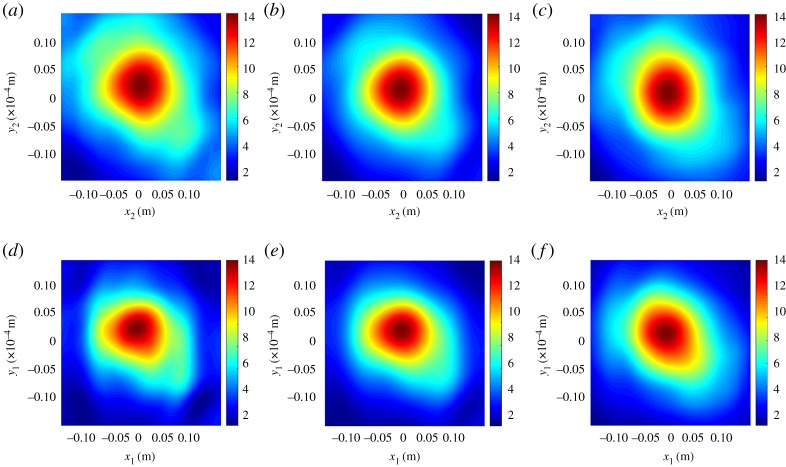
Upper row: comparison between field CFs obtained from (*a*) WF-based and (*b*) MoM-based propagators and (*c*) with measured data. Lower row: energy densities obtained from (*d*) WF-based and (*e*) MoM-based propagators and (*f*) with measured data. The observation plane correlation is taken with the reference point at (0 mm; 0 mm), and results are shown for the plane at *z* = 10 cm beyond the source. (Field correlation and energy density are in units of (A/m)^2^.)

**Figure 3.**
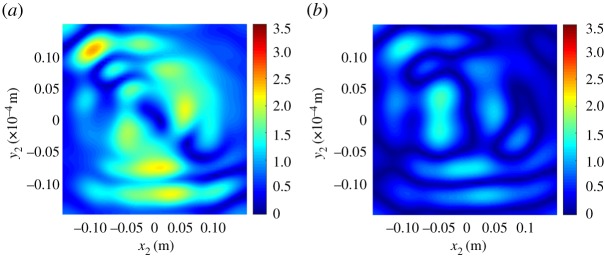
Difference between the intensity from (*a*) WF-based propagator and measured data and (*b*) MoM-based propagator and measured data. (Energy density in units of (A/m)^2^.)

Since we only consider magnetic fields oriented in the *y*-direction, the equivalent surface currents are oriented in *x*-direction. Therefore, we only need to consider the following component of the Green dyadic
3.1
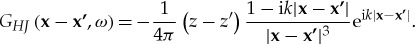
Unit pulse functions were applied as expansion functions in the discretization scheme based on the MoM
3.2
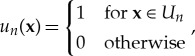


where *U*_*n*_ is the solution domain for the *n*-th basis function and Dirac delta distributions *δ*(**x**) = *δ*(*x*)*δ*(*y*) used as weighting functions. In the post-processing of the data, a four-dimensional (4D) correlation dataset *γ*_*z*_(*x*_1_, *y*_1_, *x*_2_, *y*_2_) has been calculated for each scan plane. The 4D dataset for *z* = 0.01 m is used as a source for the propagation calculation. The propagated version of the 4D dataset at *z* = 0.10 m is obtained using both methods. The computational cost for the numerical propagation of the WT in (2.21) is low, since it only involves a convolution. The MoM-based approach requires matrix multiplications for determining field correlation propagation, which is computationally very efficient. However, the computational cost of determining the impedance matrix varies depending on the chosen Green function and requires numerical integration when all near-field contributions are included. The mosaic representation described in [29] is used and the comparison between the two propagated CFs is performed on a selected square of the mosaic. This is shown in figure 2 along with resulting energy densities. There is a good agreement between the two correlation patterns both in terms of height and spreading of the correlation length. Even better agreement is found for the energy densities reported in figure 2*d*,*e*, both accurately reproducing the measured intensity in figure 2*f*. To have a better insight into the accuracy of the *approximated* WF method, we use the *exact* MoM method as a reference (where an exact WF propagator has been used in previous work [28,33]). In particular, the differences between both the propagated energy densities and the measured data have been calculated for the example in figure 2, in order to quantify the relative errors of the two methods with respect to measurements. The error plots are shown in figure 3. Both the propagated energy densities have small differences compared to the measured data with the (numerically exact) MoM method being more accurate than the approximated WF method, as expected. However, the MoM is computationally more intensive than the approximated WF and does not transparently provide information on space-angular properties of emissions as the WF does. We stress that *exact* formulations of the WF method, based on the full kernel defined in (2.20), are available [28,33] as an alternative to MoM, but these would be similarly computationally more intensive. The WF method propagates input data with the level of efficiency of a double Fourier transform. In this example, the computation time of the MoM-based propagation, with full calculation of impedances including near-field contributions, takes hours, while the computation time of the WF-based propagation takes minutes in a standard desktop computer. Since the MoM corresponds to an exact propagator, propagation inaccuracies are only related to discretization and discrepencies are likely due to measurement uncertainty. Theoretical methods and measurement techniques apply to other field components and therefore the same level of agreement is expected for other components of the CT, whence we envisage a successful reconstruction of the average Poynting vector for partially coherent stochastic vector fields.

## 4. Conclusion

This paper has presented a comparison of approaches to stochastic field measurements and modelling using WFs and MoM. It is shown that by quantifying the coherency tensor of stochastic fields, a wave-dynamical phase-space representation can be devised to extract both the energy flow vector and the local energy density. The spatial and directional properties can be extracted from the CT through the WT and the relation between the WT and Poynting vector is derived. This is then used to develop a propagation rule for the CT. A comparative study based on experimentally measured stochastic fields and propagated fields using the WF and the MoM technique is provided showing good agreement. It is shown that Heaviside anticipated many of these advanced ideas leading to the new field of statistical electromagnetics.

## Data accessibility

Datasets and codes used from both UoN and TUM to process data have been deposited: http://ggiemr-repository.engineering.nottingham.ac.uk/UoN/Wigner_MoM.

## Appendix

Using Maxwell's equations for a source- and charge-free medium in the **p** domain,

A 1

A 2

where  and **P** = [**p**, *p*_*n*_], and with *p*_*n*_ standing for the momentum along the normal direction to the surface in which in-plane components of **p** are defined, the first term in (2.15) can be written

A 3

while the second term in (2.15) is

A 4

Combining the two expressions yields

A 5

from which, unfolding the kernel inside the square brackets yields

A 6

Now, since averaging and integration commute, and using the property of the electric CT, , along with the Hermiticity of , we may write

A 7

which is consistent with the expression derived in [14, eq. (2.13)]. We can exploit the calculation further by involving the phase-space representation (2.11)

A 8

where

A 9

is implicitly a function of **p** and **q**, but not of **x** or **x**′. We may formally write this in the form (2.16), where the vector operator  acts by convolution on functions of **x** as

where

Note that the components of this operator parallel to the source plane act simply through the delta kernel

but the normal component is non-trivial.
